# Situs Inversus Partialis With a Wandering Spleen Having a Single Atrium: A Rare Tale of Survival

**DOI:** 10.7759/cureus.41860

**Published:** 2023-07-14

**Authors:** Vinit Deolikar, Rinkle R Gemnani, Keyur Saboo, Sunil Kumar, Sourya Acharya

**Affiliations:** 1 Department of Medicine, Jawaharlal Nehru Medical College, Datta Meghe Institute of Higher Education and Research, Wardha, IND; 2 Department of Internal Medicine, Jawaharlal Nehru Medical College, Datta Meghe Institute of Higher Education and Research, Wardha, IND

**Keywords:** wandering spleen, single atrium, situs inversus, congenital heart disease, case report

## Abstract

This case report of a rare condition involving situs inversus partialis, wandering spleen, and a single atrium. Situs inversus partialis is a congenital developmental defect in which the abdominal or thoracic organs are reversed to the opposite side of the body across the sagittal plane. The case report highlights the congenital developmental anomaly and the diagnostic and management complexities associated with this condition. The patient in this case has survived to the age of 24, despite the presence of a single atrium. In the existing literature, situs inversus is a known congenital condition, but partial situs inversus is less common. A wandering spleen is also a rare condition characterized by splenic hypermobility. The combination of situs inversus partialis, a wandering spleen, and a single atrium is particularly unusual and has limited reported cases. Therefore, this research contributes to the existing literature by providing a unique case report and highlighting the challenges associated with diagnosis and management in such cases.

## Introduction

The term *situs inversus* refers to a condition in which the organs of the chest and abdomen are positioned in a mirror image of normal human anatomy. Situs inversus is divided into two types, complete situs inversus, also known as situs inversus totalis, and partial situs inversus, also known as situs inversus partialis, in which some organs are inverted and others are as they are [1]. Almost all patients with situs inversus are associated with congenital heart disease, having some splenic syndromes. The incidence of these associated anomalies is 1:22,000 in the general population having a poor prognosis. The survival rate for patients beyond five years is reported to be only 5% to 13% [2,3].

The spleen is generally located in the left upper quadrant of the abdomen and is kept in position by various suspensory ligaments. A floating spleen, or wandering spleen, is an unusual condition in which there is splenic hypermobility due to elongations or maldevelopment of the spleen's suspensory ligament [2].

A single atrium, or common atrium, is a rare congenital anomaly in which there is a complete absence of the atrial septum. It leads to an arterial and venous blood mixture in the common atria, which usually causes palpitations, dyspnea, and cyanosis [3].

This case report aims to highlight the clinical presentation, diagnostic difficulties associated with partial situs inversus, and management challenges associated with this rare combination of conditions. 

## Case presentation

A 24-year-old female presented to the outpatient department of medicine with complaints of breathlessness and palpitations for several months. She was also having off-and-on epigastric pain, nausea, and vomiting. The pain was dull and aching, which the patient narrated as wandering-type from the epigastric region to the right upper quadrant of the abdomen. These episodes were of mild intensity, and every time for these symptoms, she sought treatment from a general practitioner and got relief. For persistent breathlessness and palpitations, she was referred to this hospital.

On general examination, the patient was less than average built, had a body mass index of 18 kg/m^2^, was afebrile, had a pulse rate of 120 beats per minute, a blood pressure (BP) of 90/60 mmHg, a respiratory rate of 24 breaths per minute, and had SpO2 of 85% in room air. The patient had peripheral cyanosis. The liver was palpable 7-8 cm further along the midsternal line on abdominal examination. The spleen was not palpable. A nontender palpable mass was identified in the right iliac fossa. The cardiovascular system examination was not remarkable except for peripheral cyanosis. A respiratory system examination revealed bilateral basal crackles.

Her laboratory parameters showed hemoglobin of 13.7 g/dL, white blood cells of 12,100 mm^-3^, and a platelet count of 16,000. All other parameters have been given in Table 1.

**Table 1:** Laboratory reports of the patient in reference to normal range.

Lab parameters	Observed value	Normal range
Hemoglobin	13.2 g%	13-17 g%
Mean corpuscular volume	73.9 fL	83-101 fL
Total leucocyte count	12,100 cells/mm^3^	4,000-10,000 cells/mm^3^
Platelets	16,000 mm^-3^	150,000-400,000 mm^-3^
Urea	25 mg/dL	19-43 mg/dL
Creatinine	0.5 mg/dL	0.66-1.25 mg/dL
Sodium	137 mmol/L	137-145 mmol/L
Potassium	4.3 mmol/L	3.5-5.1 mmol/L
Alkaline phosphatase	105 U/L	38-126 U/L
Alanine transaminase	16 U/L	<50 U/L
Aspartate aminotransferase	35 U/L	17-59 U/L
Albumin	4.3 g/dL	3.5-5 g/dL
Total bilirubin	0.4 mg/dL	0.2-1.3 mg/dL
Activated partial thromboplastin time	34.3 s	29.5 s
Prothrombin time	14.3 s	<20 s
International normalized ratio	1.21	1-1.5

**Figure 1:**
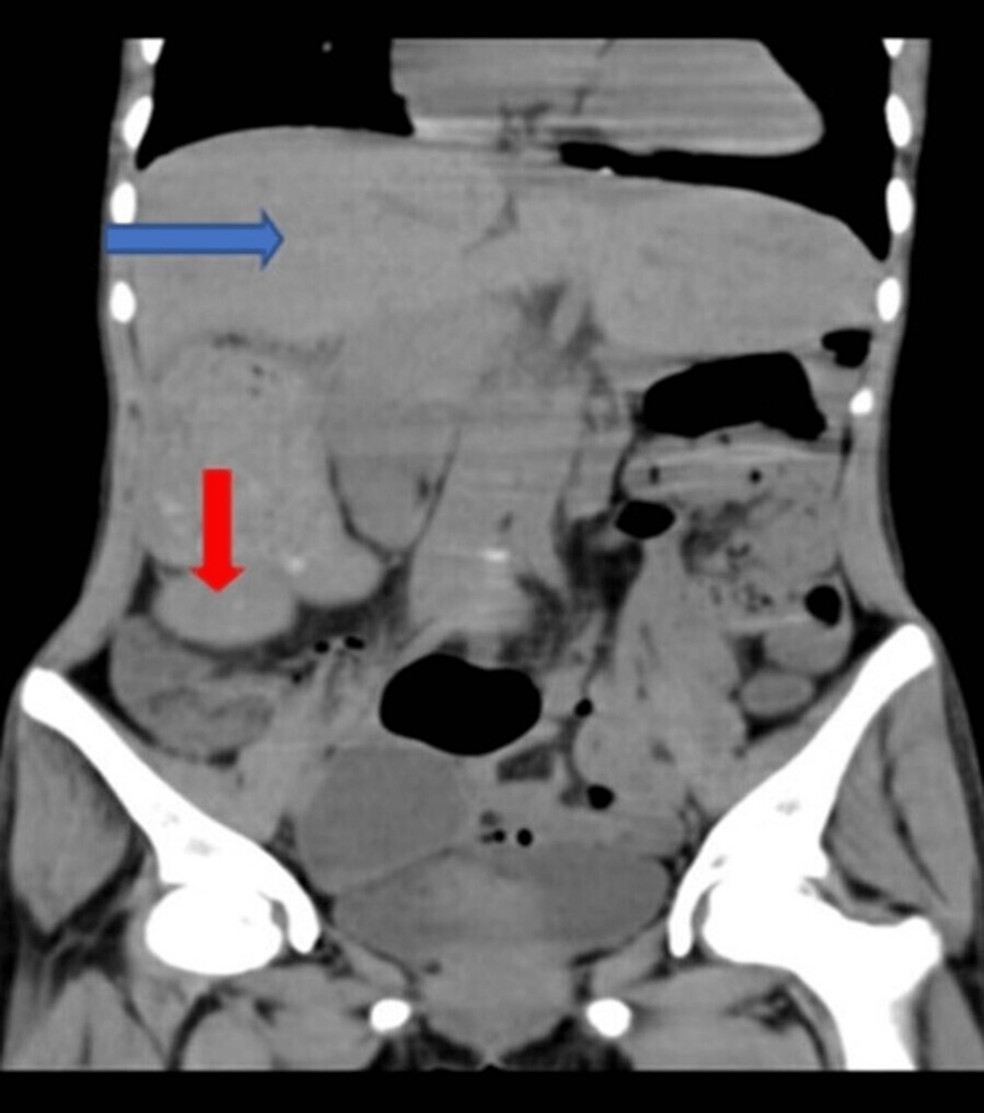
The blue arrow showing the central large liver extending from the right hypogastrium to the left hypogastrium, and the red arrow showing the splenic structure in the right iliac fossa suggestive of a wandering spleen.

**Figure 2:**
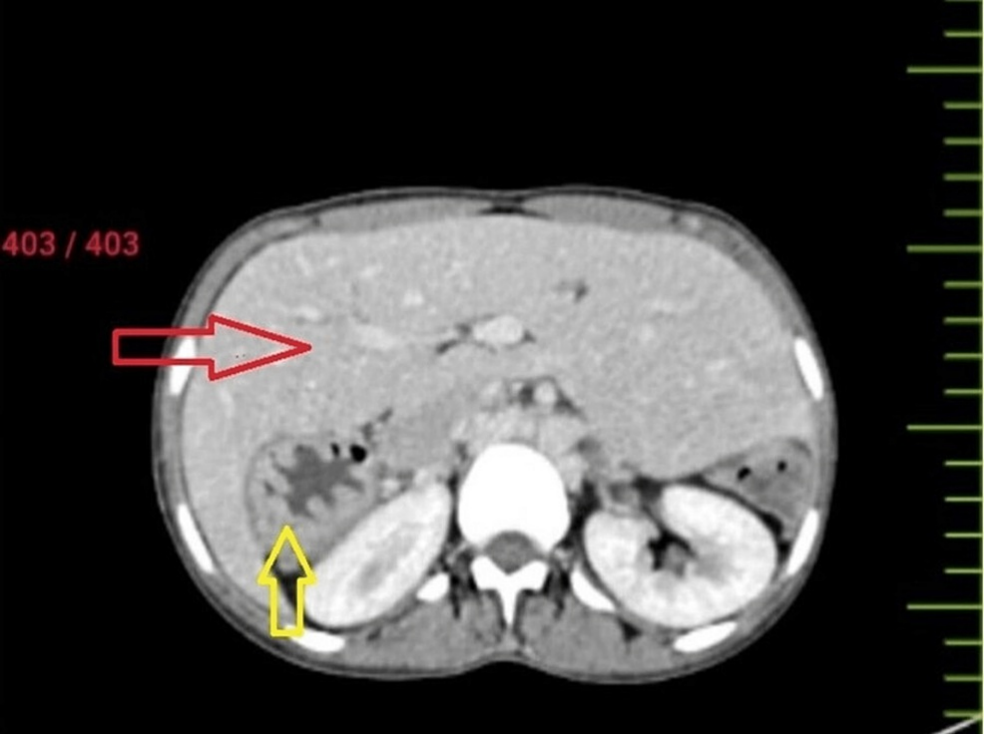
The red arrow showing the central large liver extending from the right hypogastrium to the left hypogastrium on CT angiography, and the yellow arrow showing the spleen in the right iliac fossa (wandering spleen) on CT angiography. CT, computed tomography

**Figure 3:**
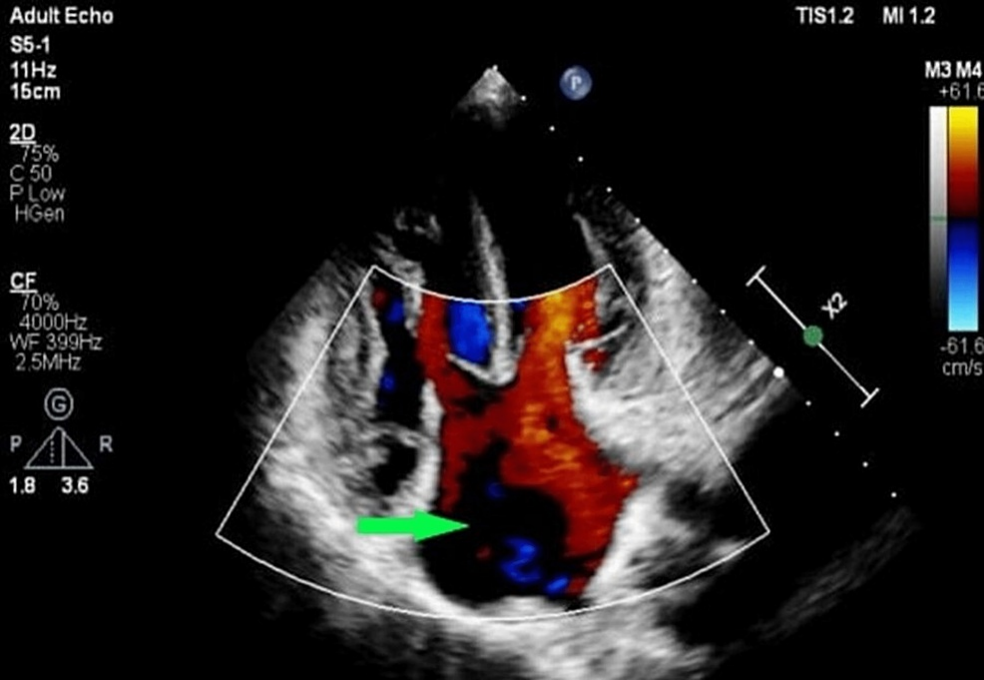
The green arrow showing a single atrium.

Ultrasonography (USG) of the abdomen and pelvis revealed a large central liver extending from the right hypogastrium to the left hypogastrium and a mass-like lesion in the right iliac fossa. The spleen was not visualized. Computed tomography (CT) angiography showed a well-defined homogeneously enhanced splenunculi structure in the right iliac region with stretched splenic vessels suggestive of the wandering spleen with an accessory splenic artery directly arising from the abdominal aorta at the T11 level and measuring 1.8 mm at the origin (Figures 1 and 2). Her two-dimensional (2D) echocardiography showed congenital heart disease, cyanotic, synchronous diagrammatic stimulation, a complete atrioventricular (AV) canal defect, an ostium primum amounting to a single atrium, an inlet ventricular septal defect with a right-to-left shunt, all four chambers were dilated, and the inferior vena cava was dilated and collapsing (Figure 3). The left ventricular ejection fraction was 30% to 35%.

The patient was managed symptomatically with oxygen therapy. A consultation with a cardiac surgeon was conducted, and the patient was counseled on non-surgical management due to complicated surgery with a poor prognosis and multiple risks. The prognosis was explained to the patient and relatives, and they were advised to follow up for further symptomatic management.

## Discussion

Situs inversus partialis is an extremely rare condition, and it is less common as compared to situs inversus totalis, both conditions are inherent as autosomal recessive defects [4,5]. Situs inversus is a morphological anomaly in which there is a reversal of visceral topography. For normal visceral anatomical structures, it requires 270° of anticlockwise rotation, whereas, in the case of situs inversus, there is 270° of clockwise rotation. Depending on the degree of malrotation, the defect can be situs inversus partialis or situs inversus totalis [1].

A wandering spleen is distinguished from the normal spleen by the absence of fixation and an excessively long splenic pedicle. As a result, the spleen can move and migrate from its typical location in the left hypochondrium. Gastrosplenic and splenorenal ligaments fix the spleen in its normal position; failure of the development of these ligaments congenitally leads to a wandering spleen [2].

The spleen grows in the dorsal mesogastrium and shifts posterolaterally to the left when the gut rotates. The lienorenal ligament is formed when the dorsal mesogastrium connects with the left kidney and the posterior abdominal wall. It contains the pancreatic tail and the splenic artery. Failure to fuse results in an excessively long pedicle.

A single atrium is a very rare condition characterized by an embryologically deficient interatrial septum. The morphology of the right atrium (crista terminalis, pectinate muscle, and atrial appendage) is in the dextral part of the common atrium, while the sinistral part has the morphology of the left atrium (smooth non-trabeculated walls and left atrial appendage) [3,6,7,8]. The prognosis of patients with a single atrium is very poor, having mortality up to 50% even in the highest facility center, as reported by Tabbah et al. [3]. 

In this case report, the patient has survived to the age of 24, despite the presence of a single atrium with neither palliative surgery nor medical evaluation. In the existing literature, situs inversus is a known congenital condition, but partial situs inversus is less common. A wandering spleen is also a rare condition characterized by splenic hypermobility. The combination of situs inversus partialis, a wandering spleen, and a single atrium is particularly unusual, and not a single case has been reported in the literature.

## Conclusions

Situs inversus partialis of the stomach with a wandering spleen having a single atrium is an extremely rare presentation. This is a congenital developmental anomaly wherein the abdominal organs or thoracic organs alone are reversed to the opposite side of the body through the sagittal plane. Partial situs inversus is of clinical importance due to the technical and diagnostic difficulty. In our case, the patient was still surviving with only mild symptoms at the age of 24 even after a single atrium. Surgical intervention was not possible as there were many risk factors. This case highlights about importance of awareness and early detection of such conditions to guide appropriate management strategies if feasible.
